# Complex population structure and haplotype patterns in the Western European honey bee from sequencing a large panel of haploid drones

**DOI:** 10.1111/1755-0998.13665

**Published:** 2022-06-27

**Authors:** David Wragg, Sonia E. Eynard, Benjamin Basso, Kamila Canale‐Tabet, Emmanuelle Labarthe, Olivier Bouchez, Kaspar Bienefeld, Małgorzata Bieńkowska, Cecilia Costa, Aleš Gregorc, Per Kryger, Melanie Parejo, M. Alice Pinto, Jean‐Pierre Bidanel, Bertrand Servin, Yves Le Conte, Alain Vignal

**Affiliations:** ^1^ GenPhySE Université de Toulouse, INRAE, INPT, INP‐ENVT Castanet Tolosan France; ^2^ Institut de l'abeille (ITSAP), UMT PrADE Avignon France; ^3^ GeT‐PlaGe, Genotoul, INRAE Castanet Tolosan France; ^4^ Bee Research Institute Hohen Neuendorf Germany; ^5^ Apiculture Division National Research Institute of Horticulture Puławy Poland; ^6^ CREA Research Centre for Agriculture and Environment Bologna Italy; ^7^ Faculty of Agriculture and Life Sciences University of Maribor Pivola Slovenia; ^8^ Department of Agroecology, Science and Technology Aarhus University Slagelse Denmark; ^9^ Agroscope, Swiss Bee Research Centre Bern Switzerland; ^10^ Centro de Investigação de Montanha (CIMO) Instituto Politécnico de Bragança Bragança Portugal; ^11^ GABI, INRAE, AgroParisTech, Université Paris‐Saclay Jouy‐en‐Josas France; ^12^ INRAE, UR 406 Abeilles et Environment, UMT PrADE Avignon France; ^13^ Roslin Institute University of Edinburgh Midlothian UK; ^14^ INRAE, UR 406 Abeilles et Environment, UMT PrADE Avignon France; ^15^ Applied Genomics and Bioinformatics, Department of Genetics, Physical Anthropology and Animal Physiology University of the Basque Country Leioa Spain

**Keywords:** genome, haplotype, honey bee, population genetics, SNP

## Abstract

Honey bee subspecies originate from specific geographical areas in Africa, Europe and the Middle East, and beekeepers interested in specific phenotypes have imported genetic material to regions outside of the bees' original range for use either in pure lines or controlled crosses. Moreover, imported drones are present in the environment and mate naturally with queens from the local subspecies. The resulting admixture complicates population genetics analyses, and population stratification can be a major problem for association studies. To better understand Western European honey bee populations, we produced a whole genome sequence and single nucleotide polymorphism (SNP) genotype data set from 870 haploid drones and demonstrate its utility for the identification of nine genetic backgrounds and various degrees of admixture in a subset of 629 samples. Five backgrounds identified correspond to subspecies, two to isolated populations on islands and two to managed populations. We also highlight several large haplotype blocks, some of which coincide with the position of centromeres. The largest is 3.6 Mb long and represents 21% of chromosome 11, with two major haplotypes corresponding to the two dominant genetic backgrounds identified. This large naturally phased data set is available as a single vcf file that can now serve as a reference for subsequent populations genomics studies in the honey bee, such as (i) selecting individuals of verified homogeneous genetic backgrounds as references, (ii) imputing genotypes from a lower‐density data set generated by an SNP‐chip or by low‐pass sequencing, or (iii) selecting SNPs compatible with the requirements of genotyping chips.

## 1 INTRODUCTION

The honey bee *Apis mellifera* comprises more than 30 subspecies, each of which is defined according to morphological, behavioural, physiological and ecological characteristics suited to their local habitat (Chen et al., 2016; Ilyasov et al., 2020; Meixner et al., 2013; Ruttner, 1988). European subspecies broadly group into two evolutionary lineages representing on one side western and northern Europe (M lineage), and on the other eastern and southern Europe (C lineage) (Ruttner, 1988). The two European M lineage subspecies are the Dark European or “black” honey bee *A. m. mellifera* and the Iberian honey bee *A. m. iberiensis*, while the C lineage subspecies include, amongst others, the Italian honey bee *A. m. ligustica* and the Carniolan honey bee *A. m. carnica* (Ilyasov et al., 2020). Prior to the involvement of apiarists, the Alps are thought to have presented a natural barrier between *A. m. mellifera* to the north, *A. m. carnica* to the southeast and *A. m. ligustica* to the southwest (Rinderer, 2013).

Before the turn of the 19th century, French honey bee populations were solely represented by the native *A. m. mellifera*, for which regional ecotypes have previously been described (Cornuet et al., 1978; Cornuet et al., 1982). However, during the 20th century, much interest arose amongst apiarists in developing hybrids between the native *A. m. mellifera* and other subspecies including *A. m. ligustica*, *A. m. carnica* and the Caucasian *A. m. caucasia* from Georgia (Cornuet et al., 1979; Fresnaye et al., 1974; Ruttner, 1988). Apiarists found the hybrids performed better than the native *A. m. mellifera* with regard to the production of honey and royal jelly, spurring further interest in the imported subspecies, which were also reported to be more docile and easier to manage (Ruttner, 1988). *A. m. ligustica* is a very popular subspecies amongst apiarists because of its adaptability to a wide range of climatic conditions, its ability to store large quantities of honey without swarming and its docile nature if disturbed (Franck et al., 2000). *A. m. ligustica* queens are frequently exported worldwide, and after the first introductions of *A. m. mellifera* and *A. m. iberica*, most of the honey bees imported over recent centuries into the New World were of Italian origin (Carpenter & Harpur, 2021; Franck et al., 2000). Apiculture involving *A. m. carnica* is also very popular amongst apiarists (Puškadija et al., 2021), in particular for further selection throughout central and western Europe (Gregorc et al., 2008; Gregorc & Lokar, 2010) given their calm temperament and higher honey yield compared to *A. m. mellifera* (Ruttner, 1988). *A. m. caucasia* is a subspecies that was also imported to France, to generate *A. m. ligustica* × *A. m. caucasia* hybrids, that were themselves crossed naturally to the *A. m. mellifera* present in the local environment. Another popular hybrid used in apiculture is the so‐called Buckfast, created and bred by Brother Adam of Buckfast Abbey in England (Adam, 1986).

Following the extensive imports of queens from “exotic” subspecies, the genetic makeup of honey bee populations in France became complex, and genetic pollution of local populations had clear phenotypic consequences such as changes in the colour of the cuticle (Cornuet et al., 1986). The increasing admixture of divergent honey bee subspecies has fostered conservationists to protect the native genetic diversity of regional ecotypes, such as *A. m. iberiensis* in Spain and Portugal, *A. m. ligustica* and *A. m. siciliana* in Italy (Fontana et al., 2018), and *A. m. mellifera* in France, Scotland and Switzerland amongst other places (De la Rúa et al., 2009; Fontana et al., 2018; Hassett et al., 2018; Parejo et al., 2016; Pinto et al., 2014). As a result of the different breeding practices, the necessity arose for a study targeted towards *A. m. mellifera* conservatories and French bee breeders specialized in rearing and selling queens. In this context, the genomic diversity project “SeqApiPop” emerged. Within this project, samples from French conservatories, from individual French breeders and from breeders' organizations were analysed, including Buckfast samples.

Traditionally, wide diversity studies have been performed using a small number of molecular markers such as microsatellites (Techer et al., 2015) or limited sets of single‐nucleotide polymorphisms (SNPs) (Henriques et al., 2018a; Parejo et al., 2018; Whitfield et al., 2006; Zayed & Whitfield, 2008), enabling population stratification, introgression and admixture levels to be characterized. However, to understand complex population admixture events, as has occurred for the managed honey bee populations in France and elsewhere, or to identify signatures of natural (Harpur et al., 2014; Henriques et al., 2018b; Parejo et al., 2020; Zayed & Whitfield, 2008) or artificial (Parejo et al., 2017; Wragg et al., 2016) selection in the genome, a much higher density of markers is required. As no high‐density SNP chip was available for the honey bee at the onset of the project, and as the honey bee genome is very small compared to most animal genomes, being only 226.5 Mb long (Wallberg et al., 2019), we used a whole‐genome sequencing approach (Harpur et al., 2014; Wallberg et al., 2014). Although the sequencing of honey bee workers has proven successful for detecting signatures of selection or admixture events (Christmas et al., 2018; Dogantzis et al., 2021; Harpur et al., 2014; Wallberg et al., 2014; Wragg et al., 2018), analysing haploid drones allows sequencing at a lower depth and with greater accuracy in variant detection, as demonstrated by studies on a limited number of samples (Henriques, et al., 2018b; Parejo et al., 2016; Wragg et al., 2016). An additional advantage of sequencing haploids is that the alleles are phased, which is invaluable for studies investigating genome dynamics such as recombination hotspots and haplotype structure. Although some insights into recombination patterns in the honey bee have been made through the analysis of drones from individual colonies (Kawakami et al., 2019; Liu et al., 2015) and linkage disequilibrium (LD)‐based approaches (Jones et al., 2019; Wallberg et al., 2015), a deep understanding of the recombination landscape, essential for fine‐scale genetic analyses, requires hundreds of phased genomes. Such “HapMap” projects have been conducted in humans and cattle, initially using SNP arrays (Bansal et al., 2007; Bovine HapMap Consortium et al., 2009) and more recently by whole‐genome sequencing as in the “1000 genome” projects (Chaisson et al., 2015; Sudmant et al., 2015).

Therefore, as the first step towards a deeper understanding of French and Western European managed honey bee populations and of their genome dynamics, and also to provide a large data set of phased genotypes from sequences aligned to the latest Amel_HAv3.1 genome assembly (Wallberg et al., 2019), we undertook the extensive sequencing of haploid drones. These data comprised samples from French conservatories and commercial breeders in addition to samples from several European countries each representing potentially pure *A. m. ligustica*, *A. m. carnica*, *A. m. mellifera* and *A. m. caucasia* populations typically imported by French breeders. Finally, *A. m. iberiensis*, the Iberian subspecies only separated from the native French *A. m. mellifera* by the natural barrier of the Pyrenees was also studied. In total, 870 samples were sequenced for SNP detection and 629 were used for a detailed genetic analysis of present‐day honey bee populations in France. The results are publicly available as a phased vcf file for high‐quality SNP markers carefully filtered against sequencing and mapping artefacts, which can be used for imputation or for selecting already genotyped reference individuals to be used in future studies.

## 2 MATERIALS AND METHODS

### 2.1 Sampling and sequencing

For the population genomics analyses, one individual drone per colony was sampled before emergence, from colonies throughout France, Spain, Germany, Switzerland, Italy, the UK, Slovenia, Poland, Denmark and China, and from a French beekeeper having imported queens from Georgia, amounting to a total of 642 samples (Figure S1, Table S1; Table 1). The robustness of the primary SNP detection by genotypegvcfs (see below) and of the filtering steps on mapping and genotype quality metrics estimated across samples were improved by increasing the size of the data set for these technical steps: a further 30 colony replicate samples, which had been collected from colonies already sampled for this study, in addition to 198 samples of similar genetic backgrounds from two other ongoing projects. Thus, although 642 colonies were included for population genomics analyses, in total 870 samples were used for SNP detection (Table S1).

**TABLE 1:** Samples used for the diversity study

Genetic type	Geographical origin^a^	Samples	Status
*A. m. carnica*	France	13	Reference (breeders)
*A. m. carnica*	Germany	18	Reference (breeders)
*A. m. carnica*	Poland	19	Reference (breeders)
*A. m. carnica*	Slovenia	20	Reference (breeders)
*A. m. carnica*	Switzerland	31	Reference (breeders)
*A. m. caucasia*	France	15	Reference (breeder)
*A. m. iberiensis*	Spain	30	Reference (beekeepers)
*A. m. ligustica*	Italy	30	Reference (breeders)
*A. m. mellifera*	Ariège, France	8	Reference (conservatory)
*A. m. mellifera*	Brittany, France	4	Reference (conservatory)
*A. m. mellifera*	Colonsay, UK	28	Reference (conservatory)
*A. m. mellifera*	Ouessant, France	41	Reference (conservatory)
*A. m. mellifera*	Porquerolles, France	15	Reference (conservatory)
*A. m. mellifera*	Savoy, France	31	Reference (conservatory)
*A. m. mellifera*	Solliès, France	14	Reference (conservatory)
Buckfast	Haut‐Rhin, France	6	Reference (breeder)
Buckfast	Switzerland	17	Reference (breeders)
Royal Jelly^b^	Breeder organization	65	Reference
Unknown	Ariège, France	12	Queen breeder
Unknown	Brittany, France	3	Breeder
Unknown	Corsica, France	44	Breeder organization
Unknown	Hautes Pyrénées, France	19	Queen breeder
Unknown	Hérault, France	7	Breeder
Unknown	Isère1, France	6	Breeder
Unknown	Isère2, France	11	Breeder
Unknown	Sarthe, France	17	Unselected apiary
Unknown	Tarn1, France	44	Queen breeder
Unknown	Tarn2, France	31	Queen breeder
Unknown	Vaucluse, France	20	Breeder
Unknown	China	10	Breeder
Unknown	Unknown	13	Breeder

*Note*: Four hundred and five samples were used as references for the genetic types commonly used in breeding in Western Europe. These include 317 samples representing five subspecies (*A. m. carnica* [*n* = 101], *A. m. ligustica* [*n* = 30], *A. m. caucasia* [*n* = 15], *A. m. iberiensis* [*n* = 30], *A. m. mellifera* [*n* = 141]), 23 samples from the Buckfast strain and 65 samples selected for royal jelly production. The remaining 237 samples were from French breeders or breeders' organizations, except 10 samples from China. All samples were collected in 2014 and 2015.

^a^ Regional geographical origins in France are indicated by their administrative “département” (see Figure S1 for locations on the map and further information on the populations).

^b^ The royal jelly samples come from several French beekeepers in Moselle, Alpes maritimes, Bouches‐du‐Rhône and Isère exchanging genetic material within the GPGR breeders' organization.

DNA was extracted from the thorax of adult bees or from pupae as described in Wragg et al. (2016). Briefly, drones were sampled at either the pupae/nymph or larval stage and stored in absolute ethanol at −20°C. DNA was extracted from the thorax or from diced whole larvae. Tissue fragments were first incubated for 3 h at 56°C in 1 ml of a solution containing 4 M urea, 10 mM Tris–HCl pH 8, 300 mM NaCl, 1% SDS, 10 mM EDTA and 0.25 mg proteinase K, after which 0.25 mg proteinase K was added and incubated overnight at 37°C. Four hundred microlitres of a saturated NaCl solution was added to the incubation, which was then gently mixed and centrifuged for 30 min at 15,000 *g*. The supernatant was treated for 5 min at room temperature with RNAse (Qiagen) and then centrifuged again, after which the DNA in the supernatant was precipitated with absolute ethanol and resuspended in 100 μl TE 10/0.1. Pair‐end sequencing was performed on Illumina HiSeq 2000, 2500 and 3000 sequencing machines with 20 samples per lane, or on a NovaSeq machine with 96 samples per lane, following the manufacturers' protocols for library reparations.

### 2.2 Mapping and genotype calling

Sequencing reads were mapped to the reference genome Amel_HAv3.1 (Wallberg et al., 2019) using bwa‐mem (version 0.7.15) (Li, 2013), and duplicates marked with picard (version 2.18.2; http://broadinstitute.github.io/picard/ ). Libraries that were sequenced in multiple runs were merged with samtools (version 1.8) (Li et al., 2009) prior to marking duplicates. Local realignment and base quality score recalibration (BQSR) were performed using gatk (version 4.1.2.) (McKenna et al., 2010), using SNPs called with gatk haplotypecaller as covariates for BQSR. Each drone was independently processed with the pipeline and genotyped independently with haplotypecaller. Although the drones sequenced are haploid, variant calling was performed using a diploid model to allow the detection and removal of SNPs for which heterozygous genotypes are called in >1% of samples, and that might have arisen for example as a result of short‐tandem repeats (STRs) or could highlight copy number variants (CNVs) in the genome. Individual gVCF files were combined with combinegvcfs and then jointly genotyped with genotypegvcfs, resulting in a single VCF file for the 870 samples containing 14,990,574 raw variants. After removing Indels with gatk selectvariants, 10,601,454 SNPs remained. Sequencing depth was estimated using mosdepth (Pedersen & Quinlan, 2018). Further details are given in https://github.com/avignal5/SeqApiPop/blob/v1.5/SeqApiPop_1_MappingCalling.md.

### 2.3 Quality filters on SNPs

The first run of filters concerns technical issues related to the sequencing and alignment steps and was therefore used for the total data set of 870 samples, to benefit from its larger size for SNP detection and validation (Figure S2). These filters included (i) strand biases and mapping quality metrics (SOR ≥3; FS ≤60 and MQ ≥40), (ii) genotyping quality metrics (QUAL >200 and QD <20), and (iii) individual SNP genotyping metrics (heterozygote calls <1%; missing genotypes <5%, allele number <4 and less than 20% of the samples with genotypes having individual GQ <10). Distribution and ECDF plots of values for all the filters used on the data set were used to select thresholds and are shown in https://github.com/avignal5/SeqApiPop/blob/v1.5/SeqApiPop_2_VcfCleanup.md.

### 2.4 Haplotype block detection, LD pruning, PCA, admixture, treemix, rfmix

Haplotype blocks were detected with plink (version 1.9) (Chang et al., 2015) using the blocks function, “‐‐blocks no‐pheno‐req no‐small‐max‐span,” with the parameter “‐‐blocks‐max‐kb 5000.” LD pruning was performed with plink using the indep‐pairwise function, with a window of 1749 SNPs, corresponding to a mean chromosome coverage of 100 kb (see Section 3), 10% overlap between windows and an LD value of 0.3. Principal component analyses (PCAs) were performed with plink and the contributions of individual SNPs to the principal components were estimated using smartpca from the eigensoft package version 7.2.1 (Patterson et al., 2006). The significance of the contribution of the SNPs to PC1 was evaluated with the r package pcadapt version 4.3.3 (Privé et al., 2020) and qvalues estimated with the r package qvalues version 2.26.0 (Storey et al., 2022). Further details are given in https://github.com/avignal5/SeqApiPop/blob/v1.5/SeqApiPop_3_LDfilterAndPCAs.md. Admixture analysis was performed with the program admixture version 1.3.0 (Alexander & Lange, 2011), with values of *K* ranging from 2 to 16. Fifty runs were performed each time using a unique random seed. The pong software (Behr et al., 2016) was used for aligning runs with different *K* values and for grouping results from runs into clustering modes, setting the similarity threshold to 0.98. Further details are given in https://github.com/avignal5/SeqApiPop/blob/v1.5/SeqApiPop_4_Admixture.md. Analyses of population migration was performed with treemix (Pickrell & Pritchard, 2012), with the option for grouping SNPs set to −*k* = 500, testing between 0 and 9 migrations and performing 100 runs per migration with a unique random seed. The optimum number of migrations was estimated with the r package optm (Fitak, R. R.: https://CRAN.R‐project.org/package=OptM) using the Evanno method provided (Evanno et al., 2005). Tree summaries for the 100 runs per migration tested were performed with dendropy (Sukumaran & Holder, 2010) and drawn with figtree version 1.4.4 (http://tree.bio.ed.ac.uk/software/figtree/). Further details are given in https://github.com/avignal5/SeqApiPop/blob/v1.5/SeqApiPop_5_TreeMix.md. Local ancestry inference and positioning of haplotype switches were performed with rfmix version 2.03‐r0 (Maples et al., 2013). Three main genetic backgrounds were considered for this analysis, corresponding to the three major groups highlighted in the PCA. Reference samples were selected as having >95% pure background. Although most diploid data were removed and the data were already phased, shapeit Version 2.904 (Delaneau et al., 2012) was run to format the vcf files for rfmix. rfmix was run using genetic maps generated from the data of Liu et al. (2015). Briefly, reads from the project SRP043350 were retrieved from the Short Read Archive (SRA) (https://www.ncbi.nlm.nih.gov/sra), aligned to the reference genome for SNP detection and recombinants were detected with the custom script find_crossing_overs.py to produce a genetic map. Further details on the rfmix analysis are given in https://github.com/avignal5/SeqApiPop/blob/v1.5/SeqApiPop_6_RFMix.md.

## 3 RESULTS

### 3.1 Sequencing and genotyping

Sequencing of the honey bee drones for the SeqApiPop diversity project began in 2014 on Illumina HiSeq instruments and some of the first samples had such low coverage that a second run (or even three in the case of OUE8) of sequencing was performed. For these samples, the resulting BAM files were merged prior to variant calling. Only four samples of the diversity project were sequenced on Novaseq instruments, for which higher sequencing depths were achieved. Therefore, to improve the robustness of the SNP detection pipeline, we included drone genome sequences from other ongoing projects using the same genetic types, which had the advantage of all being produced with a Novaseq instruments and at a higher depth. Samples sequenced with the HiSeq and NovaSeq instruments had mean sequencing depths of 12.5 ± 6.1 and 33.5 ± 10.2 respectively (Table S1, Figures S3 and S4).

Genotyping the whole data set of 870 drones with the gatk pipeline allowed the detection of 10,601,454 raw SNPs (Figure S2). Results of the subsequent filtering steps are shown on the Venn diagrams in Figures S5–S7. A total of 7,023,976 high‐quality SNPs remained after filtering. The 198 samples from the other projects and 30 within‐colony replicate samples from the present diversity project were removed from the data set for downstream analyses. Although a filter on genotyping rate ≥95% was applied in the primary filtering steps, the final filter on heterozygote calls was set to keep SNPs with up to 1% of heterozygote samples, and these remaining heterozygous genotypes were set to missing (Figure S2). After this, a final filter on missing data in samples was applied and 15 samples were removed due to the fraction of missing genotypes exceeding 10%. The final diversity data set comprised 629 drones (Table S1) and 7,012,891 SNPs, and was used for all subsequent analyses unless stated otherwise.

### 3.2 Contribution of SNPs to the variance in PCAs: detection of large haplotype blocks

**FIGURE 1:**
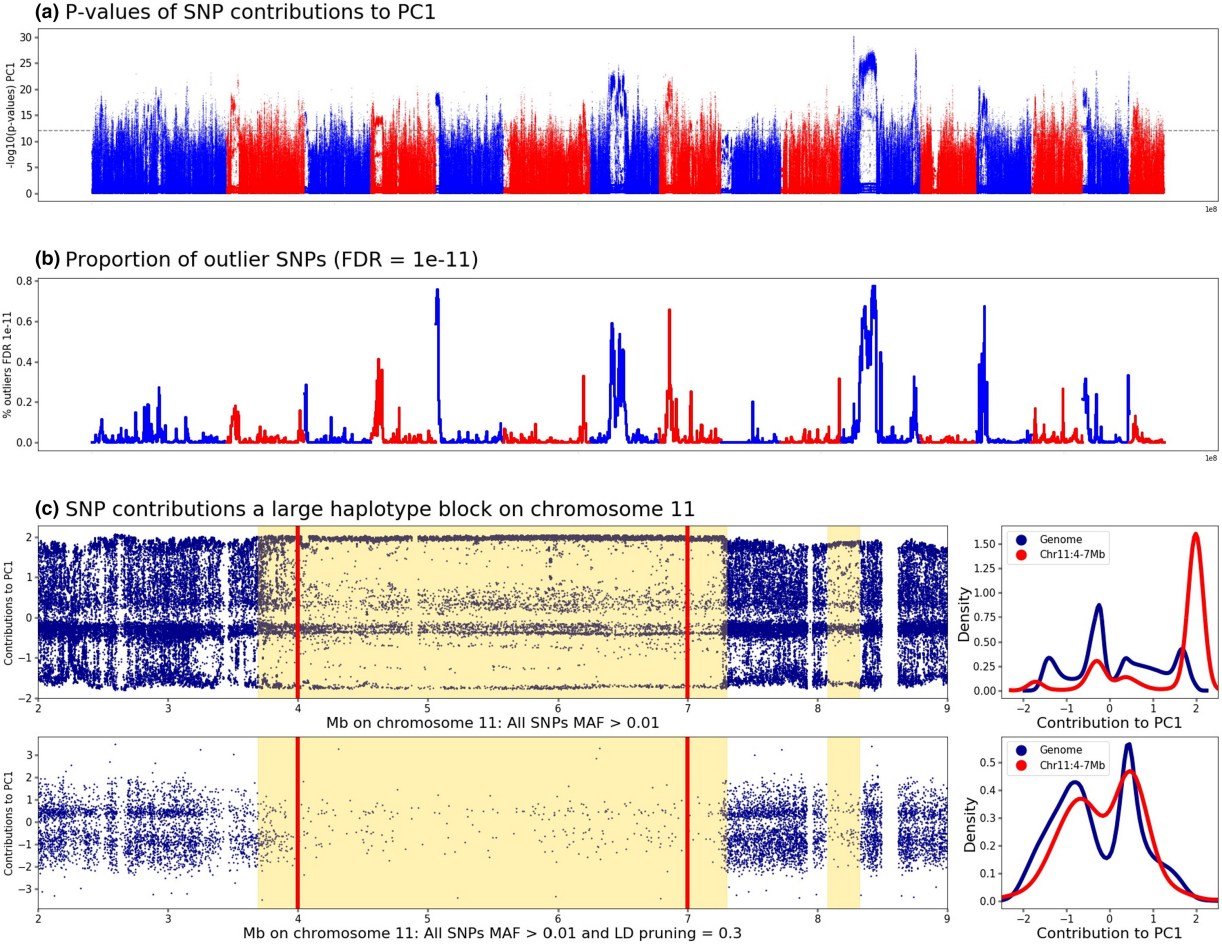
Contribution of SNPs to principal component 1: genome‐wide and in a large haplotype block on chromosome11. (a) Component‐wise *p*‐values are plotted for the correlations between PC1 and each of the 3,285,296 SNPs with MAF >1. The dashed line represents the ‐log_10_(*p*‐value) corresponding to an FDR of 10^−11^. (b) Proportion of outlier SNPs, as determined by the FDR = 10^−11^ threshold. (c) Blue points show the contribution of individual SNPs to PC1 along a 6‐Mb region of chromosome 11 containing two haplotype blocks of >3 Mb and ~200 kb (yellow backgrounds) before (top) and after (bottom) LD pruning at LD = 0.3. The LD pruning successfully eliminates the markers in the haplotype blocks and the distribution of the marker contributions approaches that of the rest of the genome, as shown in the corresponding density plots on the right

Principal component analyses, performed on the 629 samples and 7 million SNPs, resulted in a clear differentiation of three groups of samples. The first principal component, representing 10.8% of the total variance, broadly differentiates M lineage bees, *A. m. mellifera* and *A. m. iberiensis*, from the *A. m. ligustica*, *A. m. carnica* and *A. m. caucasia* bees. The second principal component, representing 3.1% of the variance, separates the O lineage *A. m. caucasia* bees from the C lineage *A. m. ligustica* and *A. m. carnica* bees (Figure S8). PC3 represents 1.2% of the variance and the remaining 626 principal components each represent 0.7% or less. When looking at the individual contributions of SNPs to the variance, we can see that only a very small proportion of the ~7 million markers contribute significantly to PC1 (red lines on Figure S9) and that this proportion is even much smaller for PCs 2 and 3. Two reasons for such a limited contribution to the variance of the majority of markers is the low informativity of markers of low minor allele frequency (MAF) and the redundancy of markers that are in strong LD. Therefore, to thin the data set, we tested the effect of several MAF filters and chose the most pertinent one for subsequent testing of various LD pruning values. The effects of these filters were estimated by inspecting the contributions of the SNPs to the principal components. The MAF filters tested showed clearly that data sets containing only SNPs with MAF >0.01 or MAF >0.05 are sufficient to allow a higher proportion of markers contributing to the PCs, with a notable increase of SNPs contributing to PC2 and PC3 (Figure S9). To avoid losing too many potential population‐specific markers present at low frequency in the data, we chose to use the lowest MAF threshold tested, leaving a data set of 3,285,296 SNPs having MAF >0.01 for subsequent analyses. On inspecting the contributions of individual SNPs to principal components along the genome, a striking feature we observe is that for several large chromosomal regions, five of which are larger than 1 Mb, a high proportion of SNPs have a significant contribution to PC1 (Figure 1a,b; and Figures S10 and S11). Such observations suggest the existence of large haplotype blocks driving differentiation along principal components, in particular PC1. To explore this further we compared these genomic regions to the haplotype blocks detected with plink (Table S2) revealing significant overlap by visual inspection (Figure S12). The largest of these blocks spans 3.6 Mb, representing 21% of chromosome 11 and close to 1.6% of the honey bee genome size (Figure 1c). Four other blocks on chromosomes 4, 7 and 9 are larger than 1 Mb (Figures S10–S12, Table S2).

### 3.3 LD filtering

Population structure and admixture analyses rely largely on the assumption that markers along the genome are independent. Indeed, markers in strong LD such as those in haplotype blocks can influence genetic structure. Therefore, we sought to investigate the impact of LD pruning on inferences of population structure. The number of SNPs used in a window for LD pruning was determined such that most windows would correspond to a physical size of 100 kb. To achieve this, we used the mode of the distribution of the number of SNPs in 100‐kb bins, which is 1749 for the data set of 3,285,296 SNPs with MAF >0.01 (Figures S13 and S14). LD pruning was thus performed with a window size of 1749 SNPs and 175‐bp (10%) overlap and various values were tested, spanning between LD .1 < *r* ^2^ ≤ .9. These various thresholds show that with LD *r* ^2^ ≤ .3 the global structure of the data set is altered, with the *A. m. iberiensis* population as the major contributor to PC2 and *A. m. caucasia* being a separate population only in PC3 (Figures S15A,B), whereas with LD *r* ^2^ > .3, the contributions to the variance in PC1 is not as widely distributed amongst subspecies (Figure S16). The effect of LD pruning on the haplotype blocks is drastic, with the few SNPs retained having a distribution of their contributions to the variance in PC1 and PC2 similar to that of the rest of the genome (Figure S17). After pruning for LD *r* ^2^ < .3, 601,945 SNPs were left in the data set, which were subsequently used in the analysis of population structure.

### 3.4 Analysis of population structure

**FIGURE 2:**
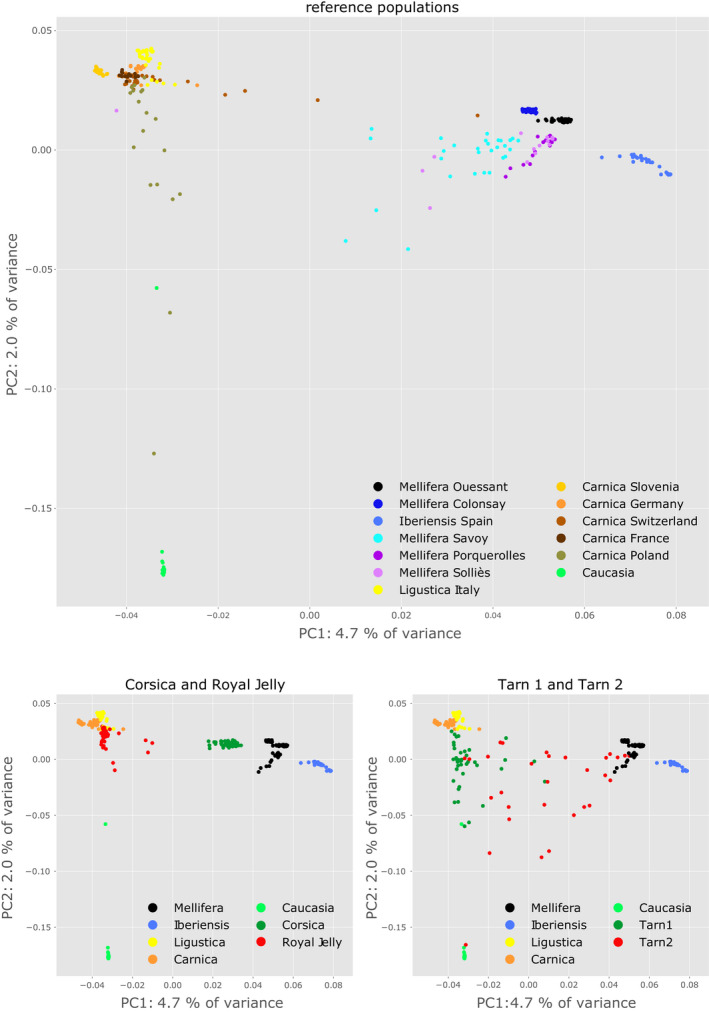
PCA on the reference populations and on a sample of representative breeder populations. The 601,945 SNPs obtained after MAF filtering and LD pruning were used. Left: reference populations only, with a colouring scheme according to their origin. Middle and left: only the reference populations with a high proportion of pure background individuals, as observed after admixture analysis, were kept and coloured according to the five subspecies. Some breeders' populations appear homogeneous, such as the honey bees selected for Royal Jelly or those from Corsica. Others are heterogeneous, such as populations Tarn1 and Tarn2, from breeders

**FIGURE 3:**
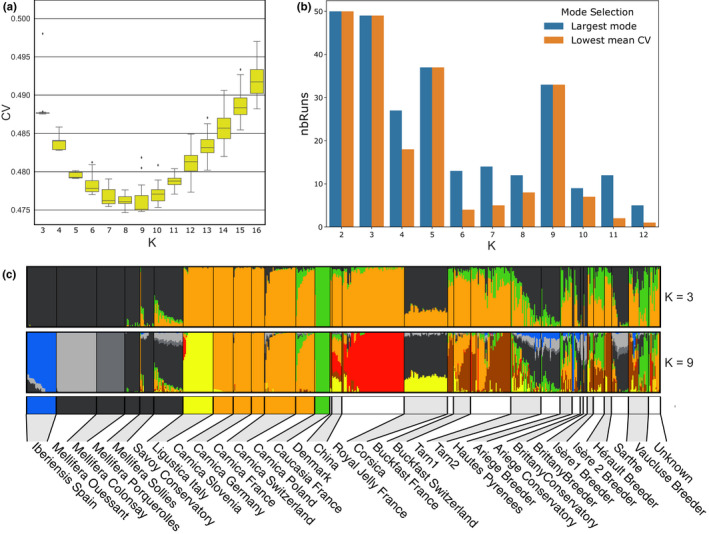
Admixture analysis. (a) Estimation of cross‐validation (CV) error for 50 runs of admixture for 3 ≤ *K* ≤ 16. (b) Major modes and modes with the lowest mean CV error for admixture runs. For each value of *K* ranging between 2 and 12, Q matrices from admixture runs were grouped by similarity in modes by using the pong software (Behr et al., 2016). Blue: number of runs in the major mode; orange: number of runs in the major or minor mode having the lowest mean CV value. Amongst the values of *K* having the lowest CV values from admixture runs, *K* = 9 stands out as having a major mode containing 33 runs out of 50 (Figure S19), which is also the mode having the lowest mean CV value from the admixture runs. For other values of *K*, such as 4, 6, 7 and 8, the major modes do not have the lowest mean CV values. (c) Admixture plots for all 629 samples for *K* = 3 (major mode containing 49 out of 50 runs) and *K* = 9 (major mode containing 33 out of 50 runs). Reference populations on the left have a colour code under the admixture plot that recapitulates their colour on the PCA plots of Figure 2; other populations are indicated with alternating grey and white colours

The PCA revealed a distinct population structure within the data. For instance, some populations from French breeding organizations, such as the royal jelly breeders' organization (GPGR: Groupement des Producteurs de Gelée Royale), and the Corsican breeder's organization (AOP Corse), appear quite homogenous (Figure 2), with GPGR samples clustering close to the *A. m. ligustica* and *A. m. carnica* reference populations, while AOP Corse samples appear as a distinct group between the C lineage *A. m. ligustica* and *A. m. carnica* on one side and the M lineage *A. m. mellifera* and *A. m. iberiensis* on the other side. Other populations from French breeders appear much less homogeneous, with individuals scattered across the whole graph (e.g. Tarn 2 on Figure 2) suggesting various degrees of admixture between the three principal genetic groups (Figure S18). To further investigate the genetic structure and the effects of human‐mediated breeding, we performed admixture analyses. Our data set consists of reference samples from 13 origins, including two islands, in addition to samples from several commercial breeders and conservatories. The genetic makeup is therefore expected to be complex and the first task was to estimate the optimal number of genetic backgrounds (*K*). We performed 50 independent runs with the admixture software for each value of 2 ≤ *K* ≤ 16 on the LD‐pruned data set, totalling 750 independent analyses. Cross‐validation (CV) error estimates of the results computed by the software are shown in Figure 3a. Results suggest that the most likely number of genetic backgrounds is 8 or 9, with *K* = 8 having runs with the lowest CV values overall, and *K* = 9 having the lowest median CV value over its 50 runs. The resulting Q matrices were jointly analysed using pong (Behr et al., 2016), where runs of each value of *K* are grouped together by similarity into modes and the mode containing the largest number of similar runs is defined as the major mode. As pong failed to find disjoint modes with the default similarity threshold of 0.97, we increased the stringency of this value to 0.98. Naturally, for low values of *K*, such as 2 or 3, most of the Q matrices are very similar and the major modes contain most runs, if not all. Typically, for *K* = 2, all 50 runs are in a single mode and for *K* = 3, the major mode contains 49 out of all 50 runs and reflects the three main groups from the PCA. Amongst the values of *K* having the lowest CV values (Figure 3a), *K* = 9 stands out as having a major mode containing 33 runs out of 50. While *K* = 8 had the lowest overall mean CV value, its major mode contained only 12 runs, indicating *K* = 9 to be a better model (Figure 3b; Table S3). As expected, the pattern observed when considering only *K* = 3 genetic backgrounds recapitulates the general pattern observed in the PCAs, in which the reference populations separate into three groups. These groups reflect the main evolutionary lineages present in the data set, being the M lineage (*A. m. mellifera* and *A. m. iberiensis*), C lineage (*A. m. ligustica*, *A. m. carnica*) and O lineage (*A. m. caucasia*). For *K* = 2, *A. m. caucasia* bees are considered as having the same genetic background as the *A. m. ligustica* and *A. m. carnica* bees, also reflecting the results from the PCA (Figure 2; Figure S19). Some admixture can be observed for a small proportion of the reference samples. For instance, the reference samples from the Savoy conservatory appear to have a small proportion of genetic background from *A. m. ligustica* and/or *A. m. carnica*, which is consistent with the PCA results (Figure 2). Likewise, the *A. m. carnica* samples from Poland have a small proportion of genetic background from *A. m. caucasia*. Finally, the *A. m. carnica* samples from Switzerland show some proportion of *A. m. mellifera* genetic background.

**FIGURE 4:**
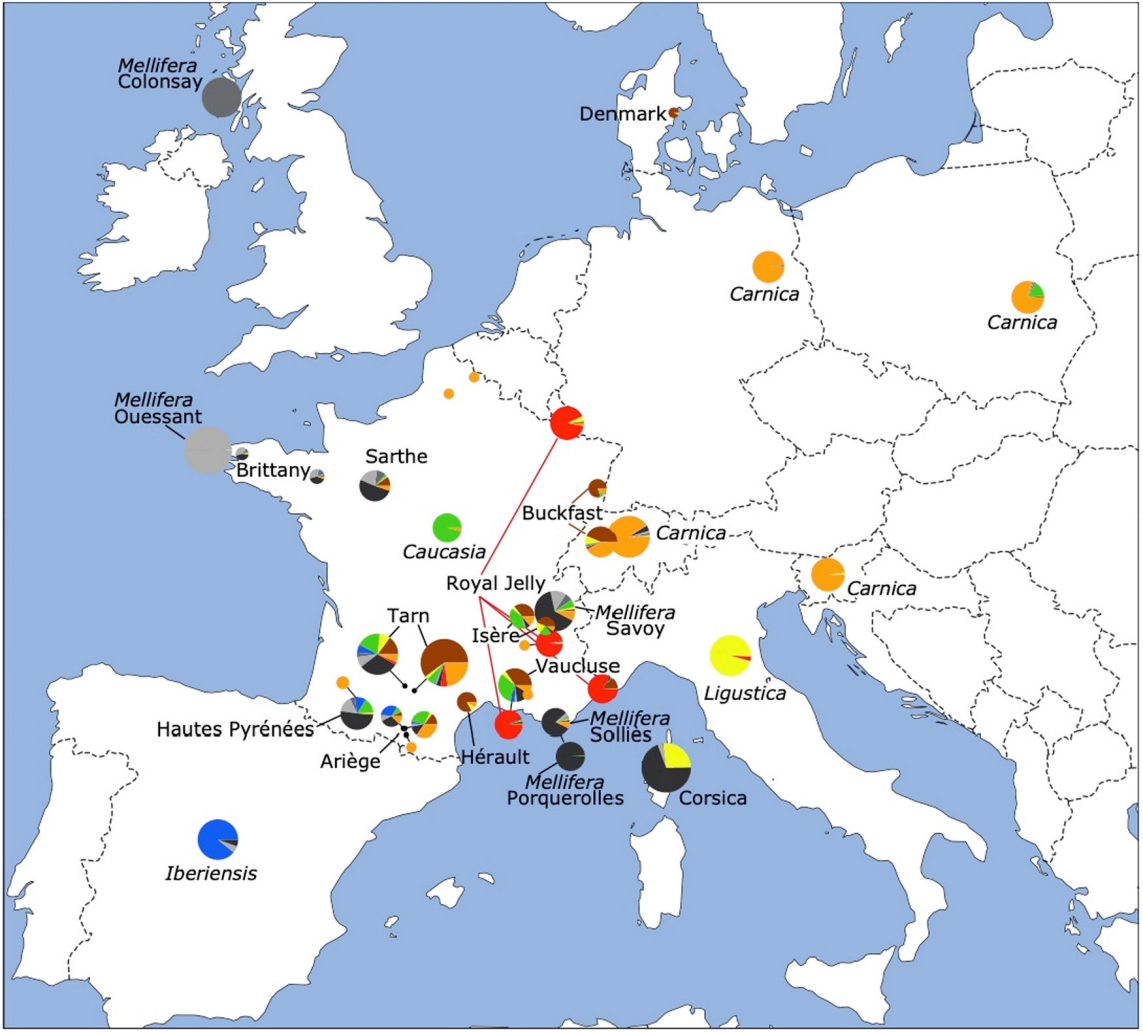
Admixture proportions and location of sample populations used in the diversity study. The size of the pie charts indicates the number of samples from a given location, with the number ranging from two samples (e.g., Denmark) to 43 samples (Corsica). Positions in France indicate the coordinates of the breeder or honey bee conservatory sampled. In other countries, reference samples are all grouped together, unless two genetic types were sampled (e.g., Switzerland). Colours in the pie charts correspond to the backgrounds found in the admixture analysis for *K* = 9, as presented in Figure 3. Reference populations for the five subspecies are indicated in italics. Two Buckfast populations in France and Switzerland are indicated, as the four breeders from the Royal Jelly breeders' organization (GPGR: Groupement des Producteurs de Gelée Royale) having provided samples

When examining the admixture pattern representing the 33 runs at *K* = 9 genetic backgrounds, the three main groups are now further subdivided. The M lineage group from the *K* = 3 backgrounds is now composed of four genetic backgrounds: *A. m. iberiensis* is separated from *A. m. mellifera*, and the *A. m. mellifera* bees are separated into three groups from mainland France, and the two islands of Ouessant and Colonsay. The other three subspecies *A. m. ligustica*, *A. m. carnica* and *A. m. caucasia* each have their own genetic background. An eighth background corresponds to the samples from the bees selected for the production of royal jelly and a ninth appears in the two populations that were noted as Buckfast bees. Although it is a major background in these two populations, a majority of samples have also a large proportion of *A. m. carnica* and, to a lesser extent, of *A. m. ligustica* backgrounds. This ninth background can also be found in other breeders' populations, principally in Hérault and Tarn1 (Figure 3c). Apart from the royal jelly population, all honey bees from breeders show high levels of admixture. Moreover, there is great variability in the genetic origins and proportions of backgrounds, even for samples coming from the same location (Figure 4). The exception is the population from Corsica, for which all samples show proportions close to 75% of *A. m. mellifera* and 25% of *A. m. ligustica* backgrounds.

### 3.5 Migrations between populations

Due to the commercial interest expressed by beekeepers for the Buckfast bees and the peculiar genetic composition observed in the Corsican population, we performed a population migration analysis with treemix (Pickrell & Pritchard, 2012). All samples having more than 80% ancestry from one of the nine backgrounds detected in the admixture analysis were selected from one of the *K* = 9 major mode Q‐matrices (Table S4), and the list supplemented with the 43 Corsican samples, making our data set composed of 10 representative groups for the European populations.

**FIGURE 5:**
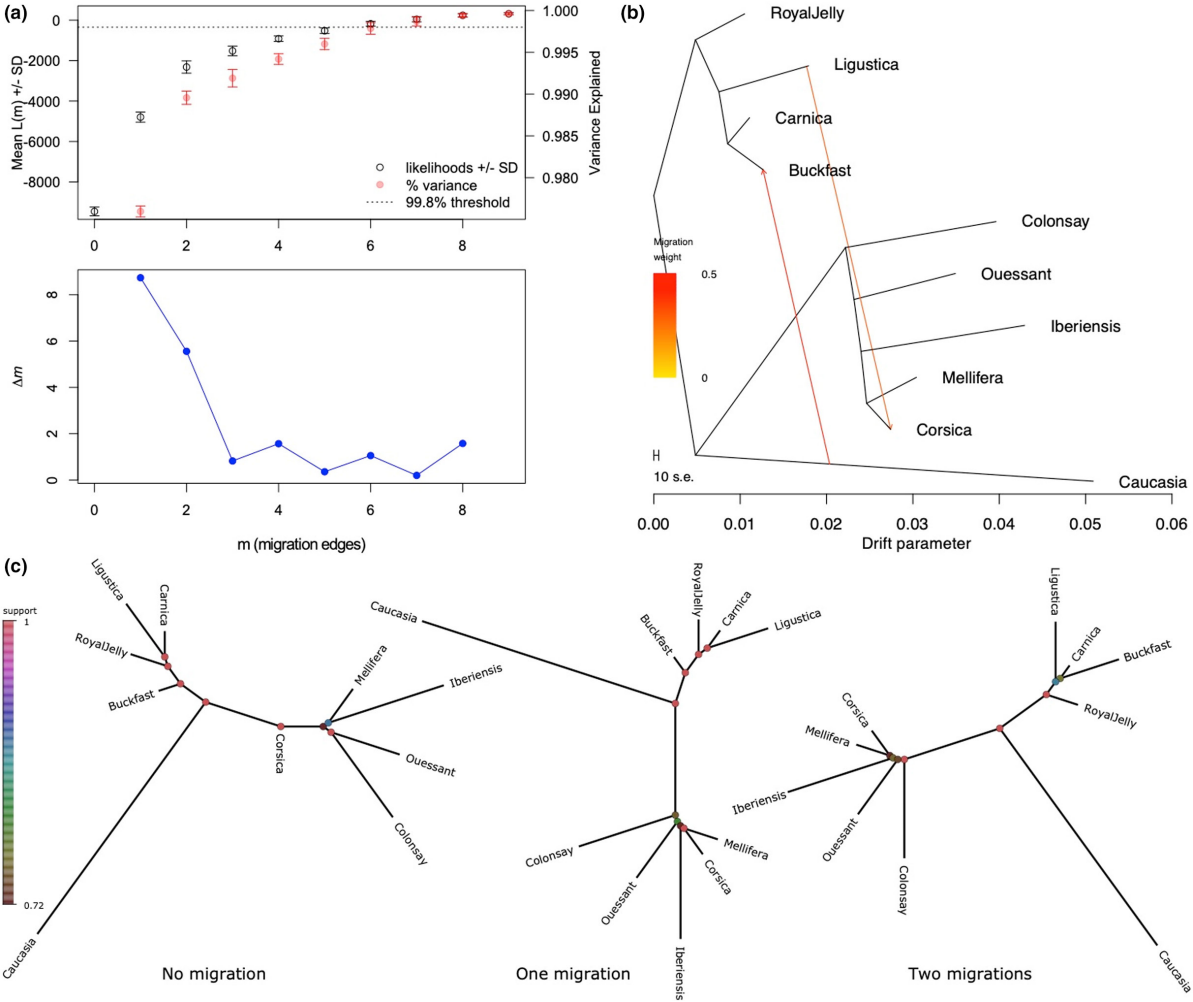
Analysis of migrations with treemix. (a) The OptM package was used to determine the optimal number of migrations between populations and backgrounds. The ∆*m* values suggest one or two migrations. (b) treemix graph selected amongst the 100 runs showing the two migrations identified. (c) Summaries of 100 trees from treemix, estimated from the 100 runs per migration with dendropy. The topologies correspond to the hypothesis of no migration (left), the Corsican population as being of the *Apis mellifera mellifera* subspecies, with a migration from an *A. m. ligustica* population (middle) or with an additional migration of *A. m. caucasia* in the Buckfast population (right). Support for the nodes is indicated by the colour, with values indicated by the scale on the left. Results suggest the original Corsican population is very similar to *A. m. mellifera* from mainland France

Estimations on the number of migrations (*m*) between the populations in the data set, based on the Evanno method (Evanno et al., 2005), return a mode of *m* = 1, strongly suggesting a single migration, and a relatively high ∆*m* value for *m* = 2 supports the existence of a second migration. The ∆*m* values for three or more migrations are close to zero, suggesting that more than two migrations between populations in the data set are unlikely (Figure 5a). For *m* = 1 the 100 treemix runs indicated a migration from *A. m. ligustica* to the Corsican population. For *m* = 2 the 100 treemix runs show the two migrations as being from *A. m. ligustica* to the Corsica population, and from *A. m. caucasia* to the Buckfast bees (Figure 5b). Summaries of the resulting trees with dendropy (Sukumaran & Holder, 2010) are shown in Figure 5c, indicating that when the two migrations are taken into account, the Corsican samples are grouped with the *A. m. mellifera* M lineage bees, and the Buckfast bees group with the *A. m. ligustica* and *A. m. carnica* C lineage bees.

### 3.6 Haplotype conservation in the admixed populations

**FIGURE 6:**
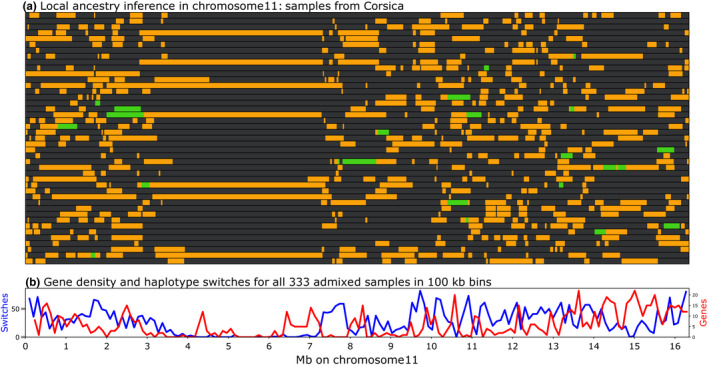
Local ancestry inference on chromosome 11 in the admixed samples from Corsica. (a) Each horizontal line represents the ancestry inference on one of the 43 individual samples from Corsica. Grey: *Apis mellifera mellifera* and *A. m. iberiensis* backgrounds; yellow: *A. m. ligustica* and *A. m. carnica* backgrounds; green: *A. m. caucasia* background. (b) Haplotype switches in all 412 admixed samples analysed. The 3‐Mb haplotype block at positions 4–7 Mb on chromosome 11 shows very little historical recombination

To investigate further the haplotype blocks detected, we performed a local ancestry inference on our data set with rfmix. Reference samples were selected as bees having >95% ancestry for a given background following the admixture analysis at *K* = 3 (Figure 3c), resulting in 131 samples for group 1, 148 for group 2 and 17 for group 3, while the remaining 333 samples formed the query data set. To perform the local ancestry inference, we constructed a genetic map from crossovers identified in the sequence data of 43 males from three colonies (Liu et al., 2015) aligned to the Amel_HAv3.1 reference genome. The results indicate that few historical recombination events have occurred in the large haplotype blocks since admixture between the subspecies. The most notable example is that of the 3.6‐Mb haplotype block between positions 3.7 and 7.3 Mb on chromosome 11, in which almost all 333 samples from the query data set show one continuous stretch for one of the three backgrounds. Only one of the 43 samples from Corsica presents two different ancestral haplotypes within this interval, with a switch from a group 1 to a group 2 haplotype at position ~4.5 Mb on chromosome 11, within the 3.6‐Mb haplotype block, whereas numerous switches can be observed on the rest of the chromosome (Figure 6a). When counting the haplotype switches detected in all 333 query samples, only 28 are located within the 3.6‐Mb haplotype block on chromosome 11, whereas other regions of the chromosome can have more than 50 switches per 100 kb (Figure 6b; and see Figure S20 for the other chromosomes). Interestingly, *LOC724287,* which is the largest gene described in the Gnomon annotation set for the Amel_HAv3.1 genome assembly, is found in this block at position 11:5,292,072–6,161,805. This gene is 869,734 bp long and encodes protein rhomboid transcript variant X2, its large size being due to intron 4, which is 596,047 bp long. However, on investigating a possible relationship between haplotype blocks and gene sizes in the honey bee genome no obvious association could be found (data not shown).

## 4 DISCUSSION

### 4.1 SNP detection in a haploid data set

Our complete data set of haploid drones is composed of 870 samples sequenced using Illumina's HiSeq and NovaSeq technologies. The results clearly show that although a few of the early sequences produced on the HiSeq are of lower depth, only 15 samples were eliminated due to the fraction of missing genotypes exceeding 10%. By contrast, the fraction of missing genotypes over the ~7 million SNPs detected was considerably lower in samples sequenced on the NovaSeq sequencing platform. Having sequenced haploids, the removal of heterozygote SNPs in individual samples is recommended to reduce the likelihood of “pseudo SNPs,” as we have shown previously that heterozygote SNPs tend to cluster together (Wragg et al., 2016) and colocate with repetitive elements (data not shown). This set of ~7 million markers can now be used as a basis for the realization of high‐density SNP chips, allowing selections of markers according to optimized spacing and to defined MAF values in the main subspecies of interest. Indeed, an important technical issue in SNP chip design is that very high SNP densities, such as found in the honey bee, can potentially cause allele dropout when genotyping, due to interference in the probe designs. Deep knowledge of SNP and indel positions will help select candidates flanked by monomorphic sequences. As an example, we have studied the overlap between our data set and the SNPs present on the 100 k SNP chip developed recently by Jones et al. (2020). The results show that of 103,270 markers originally present on this chip, 61,320 on chromosomes 1–16 are in common with our ~7 million high‐quality panel (Table S5). To investigate the effects of the high SNP density found on the honey bee genome on the potential quality of the genotyping results using the chip, we looked for additional SNPs close to each target SNP that could interfere with the genotyping process and cause allele dropout. Indeed, additional variation in the probe sequence within 50 bp flanking the target SNP will interfere with the hybridization‐based genotyping (Gershoni et al., 2022) and it is therefore common practice to have at most one extra variant within 50 bp either side of the target SNP. For SNPs with an MAF >0.2, we find that 51,178 out of 61,320 meet this criterion (16.5% of markers lost). If rare variants are considered (MAF >0.01), this number drops to 29,828 (71% of the markers lost). This suggests that SNP chips may have to be tailored towards specific populations and/or designed with prior knowledge of SNP data from population genomics studies. Conversely, for lower density chips, the spacing of markers can be optimized by taking the haplotype structure into account, thus avoiding redundancy while maintaining the highest possible level of genetic information. Another advantage of sequencing haploid samples is that the whole data set represents phased chromosomes. Notably, the present data set will be invaluable for genotype imputation in future studies using lower density genotyping, such as SNP chips or low‐pass sequencing (Gilly et al., 2019; Li et al., 2021; Wasik et al., 2021).

Although other studies have used whole genome sequencing for honey bee population genomics, these either concerned workers, thus complicating phasing (Dogantzis et al., 2021; Wallberg et al., 2014), and/or were more limited in terms of the number of samples studied (Henriques, Wallberg, et al., 2018b; Parejo et al., 2016, 2020). Moreover, to make practical use of these published results, the raw reads need to be downloaded from the short‐read archive (SRA) and aligned to the genome reference prior to detection of variants. This is a highly demanding task, in terms of both labour and computing time. In contrast, our data set is much larger, is naturally phased, and genotypes for the samples and markers can be directly selected from the vcf file provided. Moreover, our data are based on the latest version amel_hav3.1 of the genome assembly (Wallberg et al., 2019), whereas most other data sets are not, including one of the latest published, which is based on the older and less complete Amel4.5 assembly (Dogantzis et al., 2021). We believe our careful filtering steps on sequencing and alignment metrics provide a reliable set of markers and that the selection of reference samples can be done on the basis of the uniformity of genetic backgrounds, for instance by filtering on the admixture Q matrixes provided in Tables S6 and S7 on a user‐defined basis.

### 4.2 Population structure in managed honey bees

The deep understanding of European honey bee populations and of their recent admixture via imports of genetic stocks by breeders is not a simple task. Analyses of admixture events in complex population structures can be sensitive to a number of parameters and sometimes yield misleading results, especially if one or several populations have gone through a recent bottleneck (Lawson et al., 2018). PCA on all ~7 million markers indicate that our data set is structured into three main genetic types (Figure S8). The first principal component, representing 10.8% of the variance, separates two major groups corresponding respectively to subspecies from northwestern (M lineage) and southeastern Europe (C lineage). These two groups are represented by several populations, including the Savoy and Porquerolles conservatories from South‐East France on one side, and bees that are not geographically far from Italy or Slovenia on the other. This large genetic distance, despite relatively close geographical proximity of the populations, supports the hypothesis of the colonization of Europe by honey bees via distinct western and eastern routes (Estoup et al., 1995; Han et al., 2012; Ruttner, 1988; Whitfield et al., 2006), and the separation between subspecies due to the Alps forming a natural barrier preventing genetic exchange (Rinderer, 2013). Along the second principal component, representing 3.1% of the variance, the population originating from *Apis mellifera caucasia* separates from the southeastern European populations (Figure S8). Prior to investigating admixture, we pruned SNPs in LD taking care to maximize the removal of redundancy while maintaining the general structure of the data (Figure 2; Figures S15–S17).

We explored a range of *K* numbers of genetic backgrounds, running multiple iterations of each, to determine the most likely admixture pattern (Figure 3). Our results indicate that this approach is necessary to ensure the results from each *K* model are stable prior to interpretation. We observe from our admixture analyses that CV outliers within a *K* model are common. For instance, at *K* = 8, the mode with the lowest CV is only represented by eight out of 50 admixture runs, whereas the major mode has 12 runs. On examining the admixture patterns from these two modes, the major mode suggests the *A. m. mellifera* bees from conservatories on mainland France to be hybrids between bees from Ouessant and Spain, and moreover with roughly 50% of each genetic background on the same mode, the *A. m. iberiensis* background represents also 50% of the M lineage background in the bees from Corsica (Figure S19). This is unlikely given the geography of Western Europe and our knowledge of the history of the bees of Ouessant. Indeed, Ouessant is a very small island (15.6 km^2^) off the coast of western Brittany, isolated from the rest of the French honey bee population since its installation in 1987 and the prohibition of imports since 1991 mostly for sanitary reasons. In contrast, the mode with the eight runs and lowest CV presents a better separation of *A. m. mellifera* and *A. m. iberiensis*, which is also found in the major mode at *K* = 9 backgrounds. A smaller level of admixture can still be found between *A. m. mellifera* and *A. m. iberiensis*, which is quite likely to be due to the shared ancestry between these two subspecies.

The major mode at *K* = 9, represented by 33 out of 50 runs, returned the lowest mean CV value. This mode identifies mainland France *A. m. mellifera* samples as having a distinct genetic background and suggests that honey bees from Ouessant may have been re‐introduced in the mainland conservatories. This mode also identifies a distinct genetic background in French and Swiss Buckfast bees. Buckfast bees were developed by Brother Adam, and are described in page 14 of “Beekeeping at the Buckfast Abbey” as a cross performed around 1915 between “the leather‐coloured Italian bee and the old native English variety” (Brother Adam, 1986). Brother Adam also notes that the Italian bees that were imported in later years were distinct from those used in the development of the Buckfast strain. Our analysis of migrations between populations with treemix suggests that the Buckfasts in our data set were subject to introgression with genetic material from *A. m. caucasia* (Figure 5b), although the timing of this potential admixture event could not be determined. When the two migrations of *A. m. ligustica* into Corsica and *A. m. caucasia* into the Buckfast are considered, which is a likely scenario suggested by the Evanno analysis, the latter is close to *A. m. carnica*, as seen in (Figure 5b,c). Interestingly, a whole genome sequence study of Italian honey bees also suggest that the Buckfast bees are closer to *A. m. carnica* than to *A. m. ligustica* (Minozzi et al., 2021) and no proximity of the Buckfast bees with M lineage bees was found either in their study or in ours, despite the cross at the origin of the Buckfast including an old native variety. Further investigations including more Buckfast samples and additional honey bee subspecies will be needed to fully elucidate this question. The *A. m. carnica* samples from Slovenia, Germany, France, Switzerland and Poland all share the same genetic background, reflecting their identical origin, probably recent imports, relative to the history of honey bee populations.

The population of bees from Corsica comes from a breeders' organization on the island, where importation has been prohibited since the 1980s. The results show that this population has the distinct characteristic of being homogeneous in composition, despite being admixed, with all samples showing mean proportions of 75% and 25% of *A. m. mellifera* and *A. m. ligustica* backgrounds, respectively (Figures 2 and 3). The introgression of Italian bees is confirmed by the treemix migration analysis, and when this is accounted for, the Corsican samples group with *A. m. mellifera* bees from mainland France instead of being situated between the two main genetic subgroups of western and eastern European bees (Figure 2b,c). This result probably reflects the fact that Italian bees may have been imported on the Island before the ban on imports and that the population has been homogenized since then, at least within the breeders' organization. As beekeepers generally prefer the *A. m. ligustica* Italian bees over *A. m. mellifera*, it is very likely that the latter is the original population, as also suggested by Ruttner (1988). Although the hypothesis of the separation of the two subspecies on the mainland by the Alps seems appropriate (Rinderer, 2013), the situation of the Mediterranean islands in the region is not as clear. Based on physical geography alone, Corsica being at a closer distance to Italy than to France, the chances would have been greater to have originally C lineage rather than M lineage bees. Moreover, Corsica was under the control of Pisa, then fell to Genoa in 1284 and was only purchased by France in 1768. Further studies including samples from Sardinia would certainly help define the Mediterranean boundaries between the M lineage and C lineage honey bees and confirm observations based on morphology (Ruttner, 1988).

Apart from the subspecies references and the royal jelly populations, the honey bees provided by breeders are largely admixed, exhibiting high variability in background proportions—even for samples sourced from the same region. A typical example is that of the Tarn1 and Tarn2 populations, reflecting the fact that these two breeders, although situated very close to one another (<100 km), have very different genetic management strategies. The Tarn1 breeder produces Buckfast and *A. m. carnica* × *A. m. caucasia* queens by selecting within his own lines, and this is reflected in our results, in which the samples are mainly composed of Buckfast and *A. m. carnica* backgrounds, with a small amount of *A. m. caucasia*. By contrast, the Tarn2 breeder focuses principally on selecting for resistance to *Varroa destructor* and not treating his colonies with acaricides. A large proportion of *A. m. mellifera* background is present and the population is far less homogenous (Figures 2, 3, 4). This exemplifies the heterogeneity of the managed populations that can be found in France. A question that needs further investigation is the influence of the mating strategies used by the breeders, such as artificial insemination, mating stations, with drone‐producing hives to saturate the environment with the desired genetic strains, or open mating (Cao et al., 2016). Interestingly, in our data set, only three *A. m. ligustica* and all of the bees from China have some royal jelly genetic background.

Previous analyses on worldwide data sets were published, either by whole genome sequencing of workers (Chen et al., 2022; Dogantzis et al., 2021; Wallberg et al., 2014) or by sequencing the mitochondrial DNA (Tihelka et al., 2020). These were intended to understand worldwide populations, the geographical origins and migration routes of *Apis mellifera*, a topic still under debate. Our intentions here are different, being targeted towards managed populations, for which detailed knowledge of genetic makeup is essential for further work concerning traits of interest to queen breeders and beekeepers and the interaction between different subspecies present within a territory. Typically, a refined description of admixture and of its distribution along chromosomes is essential to avoid confounding effects in genome‐wide association studies (GWAS).

### 4.3 Large haplotype blocks in the honey bee genome, specific to the M and C lineages

When investigating the contribution of SNPs to variance in the PCA, we noted that several large genomic regions, up to 3.5 Mb long, in which almost all markers contributed very strongly to the first principal component, separate bees from northwestern (M lineage) and southeastern Europe (C lineage). These regions were noted to coincide with haplotype blocks detected with plink. To investigate the matter further, we performed local ancestry inference in the admixed samples with rfmix, using samples exhibiting 95% ancestry for the three main genetic backgrounds as references. A low recombination rate is confirmed by the observation of very few switches between the three main genetic backgrounds within these haplotype blocks. Interestingly, some of our regions, including the largest one detected on chromosome 11, coincide with regions of low recombination rate detected in other studies. These include an LD map produced with 30 diploid sequences from African worker bees (Wallberg et al., 2015), ancestry inference in an admixed population (Wragg et al., 2018), low‐resolution genetic maps produced by RAD or ddRAD sequencing, with microsatellite or SNP markers, ddRAD sequencing (DeLory et al., 2020; Ross et al., 2015), or higher resolution genetic maps produced by whole genome sequencing of European (Liu et al., 2015) and African subspecies (Kawakami et al., 2019).

Most of these regions coincide with the position of the centromeres such as described in the reference genome assembly, which is based primarily on the combination of the location of *Ava*I repeats, which were previously assigned to centromeres by cytogenetic analysis, and of a low GC content (Beye & Moritz, 1995; Wallberg et al., 2019). However, the *Ava*I repeats only represent a very small fraction of the centromeric regions described, with the largest one only covering 14 kb (Wallberg et al., 2019), whereas the estimate of the extent of the centromeres, based on a GC content lower than the genome average, is much larger although imprecise and supposes a similar organization as for the AT‐rich alpha‐satellite repeats in vertebrates, such as human (Altemose et al., 2022). Although in some cases the boundaries of our regions of low recombination rate coincide with the actual positioning of the centromere on the genome assembly (Wallberg et al., 2019), such as in chromosomes 5 or 8, in other instances, such as in chromosome 12, the region we define is much narrower. Due to the difficulties in interpreting banding patterns in honey bee chromosomes, the position of the centromeres is not well defined. Some evidence based on G‐ and C‐banding suggests there are four metacentric and 12 submetacentric or subtelocentric chromosomes (Hoshiba, 1984), whereas other evidence based on fluorescence in situ hybridization of a centromere probe suggests there are two metacentric, four submetacentric, two subtelocentric and eight telocentric chromosomes (Beye & Moritz, 1994). Our evidence suggests at least six chromosomes that could be telocentric or acrocentric: chromosomes 3, 5, 6, 9, 14 and 15.

Some of the haplotype blocks/regions of low recombination are large, such as representing up to 21% in the case of chromosome 11 (Figure 6). This may seem a lot, but recent findings in a complete sequencing of the human genome give a similar proportion for chromosomes 9, in which 40 Mb of satellite arrays represent 20% of the chromosome (Nurk et al., 2022). One important difference, however, is that the block on honey bee chromosome 11 contains some genes, except in the central region, whereas the satellite array described on human chromosome 9 does not. This reaffirms that our understanding of the centromere positions in the honey bee chromosomes requires refinement. The specific case of the acrocentric chromosomes in terms of gene content (Figure S20) seems to compare better to the situation described in humans, as the sequencing of the p‐arm of the five human acrocentric chromosomes has allowed the discovery of novel genes within the satellite repeat‐containing regions (Altemose et al., 2022). Centromeric DNA evolves rapidly, suggesting it goes through a genetic conflict known as the centromere drive hypothesis. This is due to the fact that in female meiosis, only one of the resulting chromosomes is included in the oocyte, leading to a competition between centromeres (Rosin & Mellone, 2017). The differentiation of centromeric regions between subspecies we observe here could be in line with this hypothesis. However, although in most species the rapidly evolving DNA sequence of centromeres is typically composed of highly repeated elements, this is not the case of the haplotype blocks found here, which seem to be associated with their respective centromeres due to a lack of recombination.

Some haplotype blocks may have another origin than centromeric DNA. For instance, genetic divergence could have been maintained by limiting recombination via the presence of structural variants such as inversions. Indeed, two of the blocks described here, between positions 4.0–5.1 and 5.8–6.9 Mb on chromosome 7, seem to coincide at least partially with two regions of haplotype divergence possibly due to inversions, detected between positions 3.9–4.3 and 6.3–7.3 Mb on the same chromosome, in a highland vs. lowland study of East African bees (Christmas et al., 2018). The slight differences in coordinates found between the two studies could be due to the fact that different version of the Amel_HAv3 assembly were used. However, if confirmed, this finding suggests that haplotype blocks differing between M lineage and C lineage bees such as found here might coincide with blocks found in other subspecies in Africa. Another study identifying the thelytoky locus (*Th*) in the South African Cape honey bee *Apis mellifera capensis* showed it was in a nonrecombining region over 100 kb long on chromosome 1, although long‐read mapping failed to detect any inversion (Aumer et al., 2019).

Given the current hypotheses on the colonization of Europe by honey bees via distinct western and eastern routes (Estoup et al., 1995; Han et al., 2012; Ruttner, 1988; Whitfield et al., 2006), it is not surprising that the haplotype blocks described here, whether or not representing centromeric regions, tend to separate mainly the M and C lineage bees. Further analyses will be necessary to define the centromeric regions more precisely and study their implication, together with the other haplotype blocks, in the subspecies structure of honey bee populations.

## 5 CONCLUSIONS

The sequencing of 870 haploid honey bee drones was shown here to be an invaluable approach for variant detection and for understanding the fine genetic makeup of a complex population having gone through multiple events of admixture. In addition, the extent of regions of extremely low recombination rate could be defined with higher precision than previously. The data set generated, based on the latest genome assembly, is a solid base for future research involving other honey bee populations and for any analyses requiring a reference set for simulations (Eynard et al., 2021), phasing or imputation.

## AUTHOR CONTRIBUTIONS

YLC, J‐PB, BB and AV designed the experiment. BB, YLC and AV coordinated colony selection, and sampling and samples were provided by KB, MB, CC, AG, PK, MP and AP. KC‐T, EL and OB performed DNA extraction, library preparation and sequencing. DW, AV, SE and BS performed the bioinformatic analyses and cowrote the manuscript. All authors read and commented on the final manuscript.

## CONFLICT OF INTERESTS

The authors declare no conflicts of interest.

6

### OPEN RESEARCH BADGES

This article has earned an Open Data Badge, for making publicly available the digitally‐shareable data necessary to reproduce the reported results. The sequence data is available at from the SequenceRead Archive (SRA) at www.ncbi.nlm.nih.gov/sra under the BioProject accessionsPRJNA311274 as part of the SeqApiPop French honey bee diversity project dataset and PRJEB16533 as part of the Swiss honey bee population and conservationgenomics project dataset. A vcf file with the filtered 7 million SNP and 870 samples is available at (https://doi.org/10.52581/zenodo.5592452) for download, together with the list of the 629 unique samples used for the diversity analysis. Scripts and supplementary description of bioinformatic analyses are available in GitHub: (https://github.com/avignal5/SeqApiPop/tree/v1.5 and a version of record of these is deposited in https://zenodo.org/record/6346402

## Supporting information


Figures S1–S20

Tables S1–S7

## Data Availability

DNA Sequences and samples metadata for this project have been deposited in the Sequence Read Archive (SRA) at www.ncbi.nlm.nih.gov/sra under the BioProject accessions PRJNA311274 as part of the SeqApiPop French honey bee diversity project dataset and PRJEB16533 as part of the Swiss honey bee population and conservation genomics project dataset. Individual SRA run and BioSample accessions for all samples are given in Table S1. A vcf file with the filtered 7 million SNP and 870 samples is available at https://doi.org/10.5281/zenodo.5592452 for download, together with the list of the 629 unique samples used for the diversity analysis. Scripts and supplementary description of bioinformatic analyses are available in GitHub: https://github.com/avignal5/SeqApiPop/tree/v1.5 and a version of record of these is deposited in https://zenodo.org/record/6346402.
